# Remarkable Luxation of the Eye

**Published:** 1859-10

**Authors:** 


### REMARKABLE LUXATION OF THE EYE.

M. Reyssie relates the following cases:

During a conflagration, the patient received the full stream from a fire-pump in his face. The column of water struck the eye-lids of the right eye with violence, thrusting them strongly backwards. Contracting under the double influence of the shock and the cold, they forcibly compressed the globe, forcing it out of its orbit by a kind of enucleation. The author saw the patient in an hour, and found the eye hanging out, retained only by its muscles, and the distended optic nerve. Its reduction was very easy. Local antiphlogistics and aperients were employed, and in the course of ten days he saw as well as before the accident. Seen thirty months after the accident, he continued quite well.—*Gaz. de Hop. Virg. Med'l Journal.*

